# Landiolol, an ultra-short acting beta-1 blocker, for preventing postoperative lung cancer recurrence: study protocol for a phase III, multicenter randomized trial with two parallel groups of patients

**DOI:** 10.1186/s13063-019-3904-4

**Published:** 2019-12-11

**Authors:** Haruko Yamamoto, Toshimitsu Hamasaki, Kaori Onda, Takashi Nojiri, Masato Aragaki, Nao Horie, Norihiro Sato, Yasuhiro Hida

**Affiliations:** 1 National Cerebral and Cardiovascular Center, 6-1, Kishibeshimmachi, Suita, Osaka 564-8565 Japan; 2 Higashiosaka City Medical Center, Higashiosaka, Osaka Japan; 3 Hokkaido University Hospital, Sapporo, Hokkaido Japan

**Keywords:** Beta-blocker, Cancer recurrence, Clinical trial, Lung cancer, Landiolol

## Abstract

**Background:**

Recurrence of cancer after curative surgery is a major problem after most cancer treatments. Increased sympathetic activity during the perioperative period could promote cancer cell invasion to blood vessels and angiogenesis, resulting in cancer metastasis. Recent studies showed that use of beta blockers can be associated with the prolonged survival of patients with cancer. The objective of this study is to evaluate the preventive effects of landiolol hydrochloride, which is an ultra-short-acting beta-1-selective blocker that has been developed in Japan, on reducing recurrence of cancer after curative surgery for patients with lung cancer.

**Methods:**

The present study is a phase III, multicenter, randomized trial with two parallel groups of patients with lung cancer, comparing surgery alone and surgery with landiolol administration for three days during the perioperative period. A total of 400 patients will be enrolled from 12 Japanese institutions. The primary endpoint is two-year relapse-free survival and overall survival after curative surgery for lung cancer. The secondary endpoints are additional treatment after recurrence of cancer, safety events, and the incidence of postoperative complications.

**Discussion:**

The principal question addressed in this trial is whether landiolol can reduce recurrence of cancer after curative surgery for lung cancer.

**Trial registration:**

Japan Registry of Clinical Trials, jRCT2011180004. Registered 17 January 2019.

## Background

Lung cancer is the most common cancer worldwide; the number of cases of lung cancer is expected to increase for some time in the future [1, 2]. It is the leading cause of cancer-related mortality in men in 87 countries and in women in 26 countries [2]. In Japan, it is the first leading cause of cancer-related mortality in men and the second of that in women. Recurrence of cancer after curative surgery is a major problem after most cancer treatments. More than 50% of patients with resectable non-small cell lung cancer (NSCLC) will have recurrence after curative surgery [3].

Increased sympathetic activity is thought to promote cancer cell invasion to blood vessels and angiogenesis, which may result in metastasis [4]. Some clinical studies report prolongation of overall survival (OS) or relapse-free survival (RFS) in patients who had used beta blockers from before diagnosis or treatment of breast, NSCLC, prostate, or ovarian cancer or malignant melanoma [5–12]. A meta-analysis study showed that use of beta blockers can be associated with the prolonged survival of patients with cancer, especially patients with early-stage cancer treated primarily with surgery [13], which contains only one study on lung cancer. Another meta-analysis study reported that non-selective use of beta-blockers is associated with improved disease-free survival (DFS) and OS in patients with ovarian cancer, improved DFS in patients with melanoma, but reduced OS in patients with lung cancer [14]. These meta-analyses have, however, major limitations such as retrospective studies, variety of beta blockers used, heterogeneity of cancer types, stages and treatment regimens, and the indications for use of beta blockers.

Landiolol hydrochloride (landiolol) is an ultra-short-acting beta-1-selective blocker that has been developed in Japan, whose pharmacokinetics and pharmacodynamics profiles are similar to esmolol [15, 16]. Because it is approved for emergency treatment of intraoperative and postoperative tachyarrhythmia, it is used for supraventricular arrhythmia including atrial fibrillation/flutter (AF) after pulmonary resection for lung cancer treatment [17, 18]. Preventive effect of landiolol for postoperative AF after heart valve surgery [19] and coronary artery bypass grafting [20] have been reported. Though the prophylactic effect of landiolol for the incidence of postoperative AF after pulmonary resection is controversial [21, 22], the safety of landiolol for perioperative use in lung resection is well-established.

The researchers who conducted the randomized controlled trial (RCT) that investigated the preventive effect of landiolol against post-pulmonary lobectomy atrial fibrillation [22] coincidentally found the tendency, though not statistically significant, of longer RFS in the group of patients who had perioperative continuous venous injection of low-dose landiolol [23].

Because prophylactic treatment for recurrence of cancer during the perioperative period is desirable, we have planned a RCT for evaluating the preventive effect of landiolol against early recurrence after curative surgery for NSCLC.

## Methods/design

### Purpose

The purpose of the present study is to evaluate the effects of landiolol hydrochloride on reducing recurrence of cancer after curative surgery in patients with lung cancer.

### Study setting

**Fig. 1:**
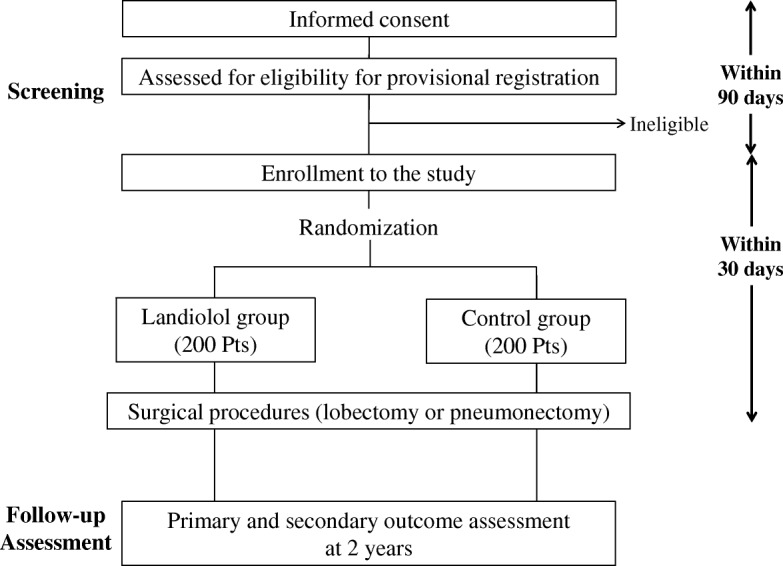
Summary of the study design

The study is an investigator-initiated, multi-institutional, confirmatory, two-arm, open-label RCT. Although landiolol is generally well-tolerated, an unblinded design was selected to safeguard the patients against possible hypotension and bradycardia. The trial design was accepted by both Hokkaido University Institutional Review Board and the Japanese regulatory agency. The flowchart of the trial is shown in Fig. 1 Additional file 1.

### Endpoints

The primary endpoints of this study are two-year RFS and OS after curative surgery for lung cancer, where RFS is defined as a patient who was alive and had no evidence of recurrence after curative surgery at the end of the follow-up period. The secondary endpoints are additional treatment after recurrence of cancer, safety events, and the incidence of postoperative complications.

### Patient selection

Inclusion and exclusion criteria are shown in Table 1. The tumors are staged according to the eighth edition of the Union for International Cancer Control TNM Classification for Lung Cancer [24].

**Table 1:** Inclusion and exclusion criteria of the study

Inclusion criteria	
Patients may enter the trial if all of the following apply:	
1. Non-small-cell lung cancer is suspected	
2. Radiologically diagnosed invasive lung tumor (a consolidation diameter > 5 mm or a whole lesion diameter > 30 mm is visualized by thin-section computed tomography)	
3. Complete lobectomy planned	
4. Complete resection including mediastinal lymph node dissection is planned	
5. No synchronous or metachronous (within five years) malignancies, except for carcinoma in situ or mucosal tumors curatively treated with local therapy	
6. Age ≥ 20 years	
7. Eastern Cooperative Oncology Group performance status 0–1	
8. Sufficient organ function (leukocyte count ≥ 1500/mL, platelet count ≥ 100,000/mL, hemoglobin ≥ 8.0 g/dL, total bilirubin ≤ 1.5 mg/dL, aspartate aminotransferase ≤ 100 IU/L, alanine aminotransferase ≤ 100 IU/L, peripheral arterial oxygen saturation on room air ≥ 92%)	
9. Written informed consent from the patient	
Exclusion criteria	
Patients may not enter the trial if any of the following apply:	
1. Active concurrent malignant diseases	
2. Mental disorders that may affect the ability or willingness to provide informed consent or abide by the study protocol	
3. Beta blocker medication necessary	
4. Beta-2 stimulator medication necessary	
5. Preoperative chemotherapy and/or radiotherapy to the target lesion	
6. Systemic steroids or immunosuppressive agent medication	
7. Uncontrollable infectious disease except for viral hepatitis	
8. Serious complications (congestive heart failure, serious coronary insufficiency, acute myocardial infarction, renal failure, liver failure, hemorrhagic gastric ulcer, intestinal paralysis, intestinal obstruction, uncontrollable diabetes mellitus, etc.)	
9. Uncontrollable autoimmune disease	
10. Contraindicated for the test drug	
- cardiogenic shock	
- diabetic ketoacidosis or metabolic acidosis	
- bradycardia including atrioventricular block and sick sinus syndrome	
- right cardiac failure due to pulmonary hypertension	
- untreated pheochromocytoma	
- history of hypersensitivity to the test drug	
11. Pregnant, lactating, or potentially pregnant	
12. Participating in another clinical trial at time of enrolment	
13. Deemed unsuitable by the primary investigator for other reasons	

### Assignment of interventions

Eligible patients will be registered and randomly assigned to either the surgery with landiolol group or the surgery alone group with a 1:1 allocation, by using permutation block randomization stratified by clinical stage (IA/IB/≥II) and site. The block sizes will not be disclosed, to ensure concealment. Due to the nature of the intervention, neither patients nor investigators can be blinded to allocation, but assessments regarding recurrence will be conducted by the assessors blind to treatment allocation.

### Trial drug

Landiolol hydrochloride (INN landiolol) is an intravenously administered, ultra-short-acting beta-1 blocker, which was developed and approved in Japan. Its half-life is approximately 4 min and its cardioselectivity (**β**1/**β**2-receptor activation) in vitro is reported to be eight times that of esmolol and 375 times that of propranolol.

### Treatment methods

Treatment flow is shown in Fig. 1. The patients enrolled in this study receive surgery alone or surgery with landiolol (group A, curative surgery with landiolol; group B, curative surgery alone). In both groups, the surgical procedures undertaken include lobectomy, pneumonectomy with systematic node dissection in open thoracotomy, or video-assisted thoracic surgery. Standard systematic node dissection (ND2) includes complete removal of the hilar and mediastinal nodes. In group A, landiolol (Ono Pharmaceutical co. ltd., Osaka, Japan) is continuously infused intravenously at 2.5 μg∙kg^−1^∙min^− 1^ for 72 h from just before the induction of anesthesia. In group B, there is no agent used in addition to curative surgery. The protocol treatment is to be stopped if curative surgery is not performed.

### Follow-up

After curative resection, the information regarding postoperative complications within 30 days following surgery is recorded and sent to the data center. All patients are followed with scheduled examinations, including a physical examination, serum biochemistry testing, chest X-ray, contrast-enhanced chest and abdominal computed tomography (CT), contrast-enhanced brain magnetic resonance imaging (MRI), and bone scintigraphy to detect postoperative recurrence for two years. Fluorodeoxyglucose positron emission tomography (FDG-PET) can replace bone scintigraphy. Chest and abdominal CT are performed every six months after surgery. Brain MRI and bone scintigraphy or FDG-PET are performed every year after surgery. At the last patient-out, all patients are surveyed for survival. The schedule of this trial is shown in Table 2.

**Table 2:** Schedule of evaluation of the study

	Entry	Treatment	1 day	3 days	7 days	30 days	180 days	1 year	2 years
Inclusion/exclusion criteria	X								
Signed consent form	X								
Medical/treatment history	X								
Vital signs	X	X	X	X	X				
Laboratory tests	X	X	X	X	X				
Respiratory function test	X								
ECG	X				X				
Body CT	X						X	X	X
Brain CT/MRI	X								X
Bone scintigraphy/ whole body PET	X								X
Surgical procedure		X							X
Medication		X	X	X	X		X	X	X
Cancer recurrence		X	X	X	X	X	X	X	X
Adverse events		X	X	X	X				

*ECG* electrocardiogram, *CT* computed tomography, *MRI* magnetic resonance imaging, *PET* positron emission tomography

### Radiological assessment

Radiographical reviews for the development of postoperative recurrence are performed by the independent central image reading board, which consists of three certified radio-oncologists. All board members are blinded to treatment allocation. All films, including chest and abdominal CT, brain MRI, and bone scintigraphy or FDG-PET scans, are reviewed regularly by the board.

### Sample size

The sample size was calculated on the basis of the primary hypothesis. The two primary endpoints, i.e. RFS and OS are evaluated by fixed-sequence procedure; RFS is first tested and OS is tested only if RFS has been statistically significant. Based on an exploratory analysis of the RCT (UMIN000007561), we hypothesized that, compared to surgery only, landiolol could produce a 50% reduction in the risk of recurrence or death (a RFS hazard ratio [HR] of 0.5), assuming 70% RFS proportion at two years in the surgery alone group. The sample size for the randomized comparison was calculated as 175 patients per group with a power of 85% by one-sided log-rank test at 2.5% level, using Collet method (a total number of required events is 75). To allow for a drop-out rate of up to around 15%, 200 patients will be recruited per arm, i.e. 400 patients in total.

With this sample size, if landiolol shows a statistically significant reduction in RFS, based on extensive Monte-Carlo simulations with bivariate survival time data including a correlation between the endpoints, the power for detecting a risk reduction in OS could be > 50% when OS HR is < 0.45, assuming 90% survival proportion at two years in the surgery alone group, by one-sided log-rank test at the level of 2.5%.

### Statistical methods

Analyses will be performed on the basis of the intention-to-treat (ITT) principle. The landiolol group will be compared against the surgery only group for all primary analysis. Patient demographic data will be summarized by groups descriptively. The primary endpoint, RFS over two years, will be estimated using the Kaplan–Meier method and compared between the two groups by the stratified log-rank test including clinical stage as a stratum. The adjusted HR with its 95% confidence interval will be calculated using a proportional hazards model. The proportional hazards assumption will be investigated graphically, with a test based on Schoenfeld residuals.

The other primary endpoint, i.e. OS, will be analyzed in the same way as RFS. To control the Type I error rate, OS will be tested only if RFS has been statistically significant. Sensitivity analysis will be performed to assess the robustness of the conclusions derived from ITT-based analysis.

Although it will be performed using standard survival methods, analysis of the primary endpoints may be not affected by withdrawals from the trial as they will be treated censored in the analysis, provided that dropping out is unrelated to prognosis. Other endpoints could be missing for patients who withdraw from the trial. The reasons for withdrawal will be reported and compared qualitatively by group. Safety data will be analyzed descriptively for the treated set, which consists of all randomized patients who receive at least one study treatment. All reported *P* values will be two-sided. The statistical analysis plan, which includes more technical and detailed elaboration of the principal features stated in the protocol, will be prepared separately and finalized before database-locking.

### Data management, monitoring, and auditing

The system for electronic data capture and data management is validated to meet the Japanese regulatory requirements. On-site monitoring including source document verification and audit are planned.

### Independent Safety Evaluation Committee

The independent safety evaluation committee (ISEC) comprises three individuals not involved in conducting the study, who have expertise in multiple disciplines, including a surgeon, an oncologist, and a biostatistician. ISEC independently reviews the reports regarding the efficacy and safety data derived from this study.

### Participating institutions

In total, 12 Japanese institutions are expected to participate in this study.

## Discussion

It remains controversial whether beta blocker may improve DFS and/or OS in various types of cancer. Most studies in the reported meta-analyses are retrospective [13, 14], a prospective controlled study should be awaited.

We chose landiolol for this multicenter RCT because it is an ultra-short-acting beta-1-selective blocker and its safety for perioperative use in lung cancer resection is well-established. Although esmolol may be more common as an ultra-short-acting beta-1 blocker in the West, it has not been approved in Japan.

Our study is designed for investigating the preventive effect of landiolol against early recurrence after curative surgery for NSCLC. The results of this study will provide clinically valuable information for future cancer treatment.

### Trial status

The protocol version number is this study is HURS001 version 3.0 (6 December 2018). Patient enrollment was begun in January 2019 and will be terminated in May 2021. The study will be completed in May 2023.

## Supplementary information

**Additional file 1.** SPIRIT checklist of this study.

## Data Availability

Not applicable.

## Abbreviations

CONSORT: Consolidated Standards of Reporting Trials
CT: Computed tomography
DSMC: Data and Safety Monitoring Committee
FAS: Full analysis set
FDG-PET: Fluorodeoxyglucose positron emission tomography
GGO: Ground-glass opacity
ITT: Intention to treat
MRI: Magnetic resonance imaging
ND2: Standard systematic node dissection
NSCLC: Non-small cell lung cancer
OS: Overall survival
RFS: Relapse-free survival
SPIRIT: Standard Protocol Items: Recommendations for Interventional Trials
